# Association Between Cardiopulmonary Bypass Time and Early Postoperative Acute Kidney Injury in Adults

**DOI:** 10.7759/cureus.115589

**Published:** 2026-09-01

**Authors:** Tanjim Hassan Maruf, Md Shafiqul Islam, Md Raisul Islam, Marina Ahmed, Md Shahidul Amin, Md Golam Sarwar, Masnoon A Noor

**Affiliations:** 1 Vascular Surgery, National Heart Foundation Hospital and Research Institute, Dhaka, BGD; 2 Cardiac Surgery, National Institute of Cardiovascular Diseases, Dhaka, BGD; 3 Cardiac Surgery, National Heart Foundation Hospital and Research Institute, Dhaka, BGD; 4 Vascular Surgery, National Institute of Cardiovascular Diseases, Dhaka, BGD; 5 Thoracic Surgery, Bangabandhu Sheikh Mujib Medical University (BSMMU), Dhaka, BGD

**Keywords:** acute kidney injury, adult cardiac surgery, cardiopulmonary bypass, cardiopulmonary bypass (cpb) duration, off-pump coronary artery bypass grafting (opcabg), on-pump cardiac surgery

## Abstract

Introduction

Cardiopulmonary bypass (CPB) has improved the safety and precision of complex cardiac surgery, but its inflammatory, embolic, and hemodynamic effects may contribute to postoperative acute kidney injury (AKI). This study explored whether longer CPB duration was associated with early postoperative AKI and related renal and clinical recovery outcomes in adult cardiac surgical patients.

Methodology

This prospective observational comparative study was conducted at the Department of Cardiac Surgery of the National Institute of Cardiovascular Diseases (NICVD) in Dhaka, Bangladesh, from July 1, 2022, to June 30, 2024. It included 100 adult cardiac patients aged 18-65 years who underwent cardiac surgery under CPB, selected by convenience sampling after ethical approval. Patients were divided into two equal groups after surgery according to recorded CPB duration: Group A included 50 patients with CPB time ≤120 minutes, while Group B included 50 patients with CPB time >120 minutes. Patients with pre-existing chronic kidney disease, kidney transplant history, emergency surgery, ejection fraction below 30%, previous cardiac surgery, or surgery without CPB were excluded.

Results

The two study groups were broadly comparable at baseline, with similar mean age (37.8±13.73 vs. 38.46±13.30 years; p=0.8), age distribution (p=0.93), gender distribution (male 58% vs. 52%; p=0.69), comorbidities, body mass index (BMI) profile, and underlying cardiac disease pattern. Smoking, hypertension, diabetes mellitus, and dyslipidemia were distributed without significant difference, while most patients in both groups had normal BMI, and isolated valvular heart disease was the commonest diagnosis. The intended difference between the groups was clearly established by CPB duration, with a mean CPB time of 93.00±11.42 minutes in Group A and 137.10±12.08 minutes in Group B (p<0.001). After surgery, patients with longer CPB time showed lower urine output at 24, 48, and 72 hours, with all differences reaching statistical significance (p<0.001). Although baseline serum creatinine was statistically insignificant (0.83±0.11 vs. 0.86±0.13 mg/dL; p=0.108), postoperative serum creatinine became significantly higher in Group B at 24, 48, and 72 hours (p<0.001). Postoperative AKI occurred in seven patients (14%) in Group A but in 23 patients (46%) in Group B, showing more than a threefold higher occurrence among patients with CPB time >120 minutes (p<0.001). Renal replacement therapy was required only in Group B in four patients (8%), and in-hospital mortality also occurred only in Group B in three patients (6%), though these differences were not statistically significant. Patients in the longer CPB group required longer mechanical ventilation (8.90±3.84 vs. 6.98±2.69 hours; p<0.001), longer ICU stay (10.15±4.68 vs. 4.87±1.58 days; p<0.001), and longer postoperative hospital stay (14.36±5.55 vs. 10.11±3.05 days; p<0.001).

Conclusion

Prolonged CPB time was significantly associated with greater early postoperative renal stress, higher AKI occurrence, and slower recovery, though the findings require cautious interpretation due to the single-center design, convenience sampling, short follow-up, and unadjusted perioperative factors.

## Introduction

Cardiopulmonary bypass (CPB) has transformed modern cardiac surgery by temporarily taking over the circulatory and respiratory functions of the heart and lungs during operations on the heart and great vessels. Since its successful introduction by John Gibbon in 1952, CPB has allowed surgeons to work in a motionless and bloodless field, making complex cardiac procedures safer and more precise. Its safe use, however, depends on the coordinated work of a specialized team, including the cardiac surgeon, perfusionist, and anesthesiologist [1-3]. Despite these advances, CPB is not a completely harmless technology; contact of blood with the artificial bypass circuit, aortic cross-clamping, cardioplegia, allogeneic blood transfusion, and surgical trauma may trigger a systemic inflammatory response, often described as the "post-pump" response. Although modern circuit design, biocompatible materials, membrane oxygenators, filters, and centrifugal pumps have reduced hemolysis, blood activation, embolic load, and postoperative complications, inflammatory and embolic effects have not been completely eliminated, and the duration of extracorporeal support remains an important determinant of postoperative outcome [4-6].

Among the major postoperative complications related to cardiac surgery, acute kidney injury (AKI) remains one of the most clinically important and potentially deleterious outcomes. Postoperative AKI was diagnosed according to the Kidney Disease: Improving Global Outcomes (KDIGO) criteria. AKI was defined as any of the following: an increase in serum creatinine by ≥0.3 mg/dL within 48 hours, an increase in serum creatinine to ≥1.5 times the baseline value within the early postoperative period, or urine output <0.5 mL/kg/hour for at least six hours. Serum creatinine and urine output were assessed preoperatively and at 24, 48, and 72 hours after surgery. Cardiac surgery-associated AKI affects a considerable proportion of patients and is linked with prolonged hospitalization, increased morbidity, and higher short- and long-term mortality [7]. The exact mechanism of AKI after cardiac surgery is complex and not fully understood, but several pathways appear to contribute. During CPB, blood contact with the gaseous interface and artificial surfaces of the bypass circuit may activate inflammatory pathways, leading to the release of proinflammatory mediators. These mediators activate leucocytes, endothelial cells, and platelets, producing systemic inflammation and organ dysfunction, including renal injury [8]. In this clinical pathway, CPB time has become an important area of investigation, as prolonged exposure to extracorporeal circulation may intensify inflammation, hemodynamic instability, embolic events, and renal hypoperfusion. Although several studies have reported an association between longer CPB duration and postoperative AKI, this relationship remains complex because renal outcome may also be influenced by operative complexity, aortic cross-clamp time, transfusion, intraoperative hemodynamic changes, diuretic or vasopressor use, nephrotoxic exposure, and baseline patient factors [5]. As the present study did not adjust for all these variables, the observed relationship should be interpreted as an association rather than evidence of an independent causal effect of CPB duration.

The rationale for the present study arises from this clinical concern, where AKI after cardiac surgery continues to burden both patients and healthcare systems through fluid overload, electrolyte disturbance, cardiovascular instability, need for renal support, prolonged ICU stay, and longer hospital admission. Since CPB duration may be partly influenced by operative planning, surgical efficiency, perfusion team coordination, and timely management of intraoperative technical or hemodynamic challenges, avoidable prolongation of CPB may represent a practical target for perioperative attention. CPB time may also reflect operative complexity; therefore, its association with postoperative AKI should be interpreted cautiously rather than as a purely modifiable causal factor. Although studies from developed and developing countries have explored this issue, evidence from our local setting remains limited despite the increasing number of cardiac patients requiring surgery under CPB. Evaluating the association between CPB time and postoperative AKI in adult cardiac surgical patients is both clinically relevant and timely, as the findings may support more careful perioperative decision-making and contribute to safer cardiac surgical practice in our healthcare context.

## Materials and methods

Study design and procedure

This prospective observational comparative study was conducted in the Department of Cardiac Surgery of the National Institute of Cardiovascular Diseases (NICVD) in Sher-E-Bangla Nagar, Dhaka, Bangladesh, from July 1, 2022, to June 30, 2024. The study population consisted of admitted adult cardiac patients aged 18-65 years who underwent elective cardiac surgery under CPB. Although the calculated sample size was 64, with 32 patients in each group, a total of 100 patients were enrolled to improve precision and strengthen group-wise comparison, with permission from the ethical committee. The participants were selected by convenience sampling and were divided into two groups according to CPB duration: Group A included 50 patients with a shorter CPB time of ≤120 minutes, while Group B included 50 patients with a longer CPB time of >120 minutes. This cutoff was selected based on previous studies evaluating postoperative AKI according to CPB duration. Patients with pre-existing chronic kidney disease, history of kidney transplant, emergency surgery, ejection fraction below 30%, previous cardiac surgery, or cardiac surgery performed without CPB were excluded from the study.

Data recording

A semi-structured questionnaire (Appendix A) and checklist (Appendix B) were first prepared in English (by the authors), incorporating the selected variables in accordance with the specific objectives of the study. Before final data collection, the pre-formed questionnaire was pre-tested on 10 patients in the Department of Cardiac Surgery of NICVD, and necessary modifications were made with the guidance of the supervisor to improve clarity, completeness, and applicability. After finalization, data were collected through face-to-face interviews with the patients and by reviewing admission records, history sheets, relevant investigation reports, and medical documents. The questionnaire recorded socio-demographic characteristics, while the checklist documented essential preoperative, intraoperative, and postoperative variables in a structured manner. Pre-set options in the checklist helped to maintain uniformity during recording, reduce observational errors, and ensure that all relevant clinical information was collected systematically throughout the study period.

Statistical analysis

Collected data were carefully checked, edited, and encoded before being entered into IBM SPSS Statistics for Windows, Version 22.0 (IBM Corp., Armonk, New York, United States), and Microsoft Excel (Microsoft Corporation, Redmond, Washington, United States) for statistical analysis. Quantitative variables were expressed as mean±standard deviation (SD) and compared using Student's t-test/Fisher's exact test for two groups. Qualitative or categorical variables were presented as frequencies and proportions, with comparisons made using the chi-squared test when appropriate. All analyses were performed at a 5% level of significance (p<0.05).

Ethical considerations

Ethical clearance for the study was obtained from the Ethical Review Committee of NICVD (approval number: 1200), and necessary permissions were also secured from the relevant departments of the institute. The study was conducted in accordance with the ethical principles of the Declaration of Helsinki. All participants were thoroughly informed about the study design and objectives prior to their inclusion. They were explicitly assured of their right to withdraw from the study at any point and for any reason, without any consequence. Written informed consent was obtained from each participant through a transparent and respectful process that clearly outlined the potential benefits and risks associated with the procedure. Confidentiality was strictly maintained, and no data were disclosed without the explicit permission of the respondents. No force was applied, and interviews were conducted only with those who willingly agreed to participate.

## Results

Table 1 shows that the age profile of the patients was almost similar between the two groups, suggesting that both shorter and longer CPB time groups were comparable in terms of age distribution. The mean age was 37.8±13.73 years in Group A patients who had a shorter CPB time of ≤120 minutes, while it was 38.46±13.30 years in Group B patients who had a longer CPB time of >120 minutes. Most of the patients in both groups belonged to the active adult age range of 30-49 years, comprising 21 patients (42%) in Group A and 24 patients (48%) in Group B. The second most common age group was 22-29 years, including 18 patients (36%) in Group A and 17 patients (34%) in Group B. Patients aged 50-59 years accounted for 10 patients (20%) in Group A and eight patients (16%) in Group B, while only one patient (2%) from each group belonged to the 60-65-year age category. The overall age distribution between the two groups was also statistically non-significant (p=0.93).

**Table 1:** Comparison of age between the two groups (N=100) Data are presented as mean±standard deviation and frequency and percentage. The independent-samples t-test was used for continuous variables.

Age in years	Group A (n=50)	Group B (n=50)	t-value	P-value
22-29	18 (36%)	17 (34%)	0.24	0.93
30-49	21 (42%)	24 (48%)		
50-59	10 (20%)	8 (16%)		
60-65	1 (2%)	1 (2%)		
Mean±SD	37.8±13.73	38.46±13.30		

Table 2 presents the gender distribution of the study patients between the two CPB duration groups, showing that males were slightly predominant in both groups. In Group A, among patients with shorter CPB time, 29 out of 50 patients (58%) were male, and 21 patients (42%) were female, while in Group B, among patients with longer CPB time, 26 out of 50 patients (52%) were male, and 24 patients (48%) were female. Although Group A had a marginally higher proportion of male patients than Group B, the difference was small, with only a 6% higher male representation in Group A and a corresponding 6% higher female representation in Group B. The p-value of 0.69 indicates that this difference was not statistically significant.

**Table 2:** Comparison of patients by gender between the two groups (N=100) Data are presented as frequency and percentage. The chi-squared test was used for categorical variables.

Gender	Group A (n=50) f (%)	Group B (n=50) f (%)	χ² value	P-value
Male	29 (58%)	26 (52%)	0.36	0.69
Female	21 (42%)	24 (48%)		

Table 3 illustrates the distribution of major comorbidities among the study subjects and demonstrates that both groups were broadly comparable at baseline. Smoking was present in 16 patients (32%) in Group A and 14 patients (28%) in Group B, with no statistically significant difference (p=0.83). Hypertension was observed in 22 patients (44%) in Group A and 20 patients (40%) in Group B, again showing a similar distribution between the groups (p=0.84). Diabetes mellitus was almost equally distributed, affecting 19 patients (38%) in Group A and 20 patients (40%) in Group B, with a p-value of 1.0, indicating no meaningful difference. Dyslipidemia was found in 12 patients (24%) in Group A and nine patients (18%) in Group B, but this difference was also not statistically significant (p=0.62).

**Table 3:** Comorbidities of the study subjects between the two groups (N=100) Data are presented as frequency and percentage. The chi-squared test was used for categorical variables.

Comorbidities	Group A (n=50) f (%)	Group B (n=50) f (%)	χ² value	P-value
Smoking	16 (32%)	14 (28%)	0.19	0.83
Hypertension	22 (44%)	20 (40%)	0.16	0.84
Diabetes mellitus	19 (38%)	20 (40%)	0.04	1.0
Dyslipidemia	12 (24%)	9 (18%)	0.54	0.62

Table 4 demonstrates the preoperative anthropometric profile of the study subjects according to body mass index (BMI), showing that the nutritional and body weight status of patients was almost identical between the two groups before surgery. In Group A, among patients with shorter CPB time, five patients (10%) had BMI <18.5 kg/m², 36 patients (72%) had BMI between 18.5 and 24.9 kg/m², and nine patients (18%) had BMI between 25 and 29.9 kg/m². In Group B, among patients with longer CPB time, five patients (10%) were underweight with BMI <18.5 kg/m², 35 patients (70%) were within the 18.5-24.9 kg/m² range, and 10 patients (20%) were in the 25-29.9 kg/m² category. The largest proportion of patients in both groups belonged to the normal BMI range, accounting for 72% in Group A and 70% in Group B, while the underweight category was exactly the same in both groups at 10%. The p-value of 1.0 indicates that there was no statistically significant difference in BMI distribution between the two groups.

**Table 4:** Comparison of preoperative anthropometric findings between the two groups (N=100) Data are presented as frequency and percentage. Fisher's exact test was used for categorical variables.

Body mass index (kg/m²)	Group A (n=50) f (%)	Group B (n=50) f (%)	P-value
<18.5	5 (10%)	5 (10%)	1.0
18.5-24.9	36 (72%)	35 (70%)	
25-29.9	9 (18%)	10 (20%)	

Table 5 outlines the distribution of underlying cardiac diseases among the study subjects and shows that the pattern of disease was highly comparable between the two CPB duration groups. In Group A, among patients with shorter CPB time, the most common diagnosis was isolated valvular heart disease, found in 23 patients (46%), followed by congenital heart disease in 18 patients (36%), coronary artery disease in three patients (6%), and other mixed procedures in six patients (12%). A similar pattern was observed in Group B, where 22 patients (44%) had isolated valvular heart disease, 20 patients (40%) had congenital heart disease, three patients (6%) had coronary artery disease, and five patients (10%) underwent other mixed procedures. The proportion of isolated valvular heart disease differed by only 2% between the groups, coronary artery disease was exactly equal at 6% in both groups, and mixed procedures were also closely distributed, with 12% in Group A and 10% in Group B. The p-value of 0.97 indicates that there was no statistically significant difference in disease pattern between the groups.

**Table 5:** Comparison of type of disease between the two groups (N=100) Data are presented as frequency and percentage. Fisher's exact test was used for categorical variables.

Type of disease	Group A (n=50) f (%)	Group B (n=50) f (%)	P-value
Isolated valvular heart disease	23 (46%)	22 (44%)	0.97
Congenital heart disease	18 (36%)	20 (40%)	
Coronary artery disease	3 (6%)	3 (6%)	
Others (mixed procedure)	6 (12%)	5 (10%)	

Table 6 presents the comparison of CPB time between the two study groups and clearly demonstrates the intended separation of patients according to CPB duration. In Group A, patients who had a shorter CPB time of ≤120 minutes showed a mean CPB duration of 93.00±11.42 minutes, while in Group B, patients who had a longer CPB time of >120 minutes had a markedly higher mean CPB duration of 137.10±12.08 minutes. The mean difference between the two groups was approximately 44.10 minutes.

**Table 6:** Comparison of cardiopulmonary bypass time between the two groups (N=100) Data are presented as mean±standard deviation. The independent-samples t-test was used for continuous variables.

Per-operative attributes	Group A (n=50) mean±SD	Group B (n=50) mean±SD	t-value	P-value
Cardiopulmonary bypass time (minutes)	93.00±11.42	137.10±12.08	18.76	<0.001

Table 7 shows the postoperative urine output pattern between the two groups and reveals that patients with shorter CPB time maintained consistently higher urine output during the early postoperative period compared with patients with longer CPB time. At 24 hours after surgery, the mean urine output was 1.28±0.18 mL/kg/hour in Group A and 1.13±0.15 mL/kg/hour in Group B, showing a difference of 0.15 mL/kg/hour, which was statistically significant (p<0.001). At 48 hours, urine output remained higher in Group A, with a mean value of 1.31±0.20 mL/kg/hour, compared with 1.19±0.18 mL/kg/hour in Group B, again demonstrating a significant difference (p<0.001). By 72 hours, both groups showed a slight decline in urine output, but Group A still maintained a higher mean output of 1.18±0.14 mL/kg/hour, whereas Group B recorded 1.10±0.11 mL/kg/hour, and the difference remained statistically significant (p<0.001).

**Table 7:** Comparison of postoperative urine output between the two groups (N=100) Data are presented as mean±standard deviation. The independent-samples t-test was used for continuous variables.

Postoperative urine output (mL/kg/hour)	Group A (n=50) mean±SD	Group B (n=50) mean±SD	t-value	P-value
At 24 hours	1.28±0.18	1.13±0.15	-4.53	<0.001
At 48 hours	1.31±0.20	1.19±0.18	-3.15	<0.001
At 72 hours	1.18±0.14	1.10±0.11	-3.18	<0.001

Table 8 presents the perioperative serum creatinine profile of the study subjects and shows a clear postoperative divergence between the two CPB duration groups. At baseline, before surgery, serum creatinine was almost similar between the groups, with a mean value of 0.83±0.11 mg/dL in Group A and 0.86±0.13 mg/dL in Group B, and this difference was not statistically significant (p=0.108), indicating comparable preoperative renal status. After surgery, serum creatinine increased more prominently in patients with longer CPB time. At 24 hours, the mean serum creatinine was 0.90±0.30 mg/dL in Group A compared with 1.22±0.56 mg/dL in Group B, showing a statistically significant difference (p<0.001). At 48 hours, the difference became more evident, with Group A showing 0.92±0.39 mg/dL and Group B showing 1.30±0.59 mg/dL, again statistically significant (p<0.001). At 72 hours, serum creatinine slightly decreased in both groups but remained higher in Group B, with 0.88±0.30 mg/dL in Group A and 1.24±0.47 mg/dL in Group B (p<0.001).

**Table 8:** Comparison of perioperative serum creatinine between the two groups (N=100) Data are presented as mean±standard deviation. The independent-samples t-test was used for continuous variables.

Perioperative serum creatinine (mg/dL)	Group A (n=50) mean±SD	Group B (n=50) mean±SD	t-value	P-value
Baseline (preoperative)	0.83±0.11	0.86±0.13	1.24	0.108
24 hours after surgery	0.90±0.30	1.22±0.56	3.56	<0.001
48 hours after surgery	0.92±0.39	1.30±0.59	3.8	<0.001
72 hours after surgery	0.88±0.30	1.24±0.47	4.56	<0.001

Table 9 provides the occurrence of postoperative AKI between the two study groups and shows a marked difference according to CPB duration. In Group A, where patients had a shorter CPB time of ≤120 minutes, postoperative AKI developed in seven out of 50 patients (14%), while 43 patients (86%) did not develop AKI. In Group B, where patients had a longer CPB time of >120 minutes, AKI occurred in 23 out of 50 patients (46%), and only 27 patients (54%) remained free from AKI. The occurrence of AKI was more than three times higher in Group B compared with Group A, with an absolute difference of 32% between the groups. This difference was statistically highly significant (p<0.001)

**Table 9:** Comparison of postoperative acute kidney injury between the two groups (N=100) Data are presented as frequency and percentage. The chi-squared test was used for categorical variables.

Postoperative acute kidney injury	Group A (n=50) f (%)	Group B (n=50) f (%)	χ² value	P-value
Acute kidney injury	7 (14%)	23 (46%)	12.19	<0.001
Non-acute kidney injury	43 (86%)	27 (54%)		

Table 10 shows the postoperative clinical outcomes of the study subjects and shows that patients with longer CPB time experienced a comparatively more difficult postoperative course. In Group A, none of the patients required renal replacement therapy, while in Group B, four patients (8%) required renal replacement therapy. In-hospital mortality was absent in Group A, whereas three patients (6%) died in Group B, but this difference also did not reach statistical significance (p=0.24). The continuous postoperative outcome variables showed clear and statistically significant differences between the groups. The mean mechanical ventilation time was 6.98±2.69 hours in Group A compared with 8.90±3.84 hours in Group B, indicating that patients with longer CPB time required a longer period of ventilatory support (p<0.001). The duration of ICU stay was also markedly longer in Group B, with a mean of 10.15±4.68 days, compared with 4.87±1.58 days in Group A (p<0.001). Postoperative hospital stay was prolonged in Group B, where patients stayed for 14.36±5.55 days, while Group A patients stayed for 10.11±3.05 days (p<0.001).

**Table 10:** Comparison of postoperative clinical outcome between the two groups (N=100) Data are presented as mean±standard deviation or frequency and percentage. The independent-samples t-test was used for continuous variables, and the chi-squared test or Fisher's exact test was used for categorical variables, as appropriate.

Postoperative outcome	Group A (n=50)	Group B (n=50)	χ² value	P-value
Need for renal replacement therapy	0 (0%)	4 (8%)	-	0.12
In-hospital mortality	0 (0%)	3 (6%)	-	0.24
Mechanical ventilation time (hours)	6.98±2.69	8.90±3.84	2.89	<0.001
ICU stay (days)	4.87±1.58	10.15±4.68	7.56	<0.001
Postoperative hospital stay (days)	10.11±3.05	14.36±5.55	4.74	<0.001

## Discussion

This study explored the association between CPB duration and postoperative AKI among adult patients undergoing cardiac surgery. The present findings were interpreted in relation to previous studies that evaluated the association between CPB duration and postoperative renal outcomes. The age distribution was almost identical between the two CPB duration groups, as the mean age was 37.8±13.73 years in Group A and 38.46±13.30 years in Group B, with no statistically significant difference (p=0.8). The age category distribution was also comparable (p=0.93), with most patients belonging to the 30-49-year group in both Group A (21 patients, 42%) and Group B (24 patients, 48%). This finding indicates that age was well balanced between patients with shorter and longer CPB time in the present study. In comparison, one study reported an older study population, where the mean age was 49.21±8.89 years among patients with shorter CPB time and 52.15±9.44 years among patients with longer CPB time [9]. Although both studies showed a similar pattern of age comparability between shorter and longer CPB groups, the patients in that study were approximately 11-14 years older than those in the present study. This difference may reflect variation in patient demographics, disease pattern, timing of surgical intervention, or regional referral characteristics; however, in both studies, age did not appear to create a major baseline imbalance between the comparison groups.

Gender distribution was broadly balanced between the two CPB duration groups, with 29 males (58%) and 21 females (42%) in Group A and 26 males (52%) and 24 females (48%) in Group B, showing no statistically significant difference (p=0.69); this indicates that sex was unlikely to create a major baseline imbalance between patients with shorter and longer CPB time. The same previous comparing study reported a more male-dominant cohort in their study of 182 on-pump coronary artery bypass grafting (CABG) patients, where 133 patients (73.08%) were male and 49 patients (26.92%) were female; their patients were divided equally into 91 patients with CPB <90 minutes and 91 patients with CPB ≥90 minutes, and the study focused on postoperative in-hospital outcomes after CABG, including renal injury and mortality [9]. Another study also showed male predominance in a cardiovascular surgery cohort of 144 patients, where 95 patients (66%) were male; among patients who developed AKI, 38 of 59 (64.4%) were male, and among non-AKI patients, 57 of 85 (67%) were male, with no significant gender difference (p=0.741) [10]. In their subgroup of patients with CPB duration >120 minutes, 55 of 85 patients (64.7%) were male, including 31 of 46 AKI patients (67.4%) and 24 of 39 non-AKI patients (61.5%), again without a significant gender difference (p=0.574). A matched-pair analysis of 1428 CABG patients deliberately matched 714 off-pump CABG patients with 714 conventional on-pump CABG patients using key baseline factors including age, gender, diabetes, renal function, and ejection fraction; as a result, the female count was exactly the same in both groups, with 165 females in the off-pump coronary artery bypass (OPCAB) group and 165 females in the conventional coronary artery bypass (CCB) group (p=1.0), showing perfect gender balance by study design [11].

Postoperative AKI was markedly higher among patients with longer CPB time, as it was shown that AKI developed in only seven patients (14%) in Group A, who had a CPB time of ≤120 minutes, compared with 23 patients (46%) in Group B, who had a CPB time of >120 minutes, and this difference was statistically highly significant (p<0.001). This association should be interpreted within the broader intraoperative context, as longer CPB time may also reflect greater operative complexity, longer aortic cross-clamp time, higher transfusion requirement, hemodynamic fluctuation, vasopressor use, diuretic exposure, or other technical factors that were not fully adjusted in the present analysis. This finding is closely aligned with the observation of the study from Taiwan, which was conducted on 144 patients undergoing cardiovascular surgery with CPB and divided them according to CPB duration; they found that patients with CPB time more than 120 minutes had a substantially higher occurrence of postoperative AKI than those with shorter CPB time, with AKI rates of 54.1% versus 22.03%. Their work also added a biomarker-focused dimension, as they evaluated urinary liver-type fatty acid-binding protein (L-FABP) in postoperative urine samples and reported that it showed favorable performance in discriminating the onset of AKI within seven days, suggesting that prolonged CPB time not only increased renal risk but could also be linked with early biomarker-detectable renal injury. Similar findings were also reported by a study, where postoperative AKI was more frequent among patients exposed to longer CPB duration, supporting the concept that extended extracorporeal circulation contributes to renal vulnerability after cardiac surgery [12].

A larger study investigated the relationship between CPB duration and postoperative acute renal failure and found that postoperative AKI occurred in 72 patients (2%) overall; among 100 patients with estimated glomerular filtration rate below 30 mL/min/1.73 m², 22% developed acute renal failure, and 16 patients required dialysis [10]. They further reported that the 30-day mortality was markedly higher among patients with acute renal failure, reaching 31%, compared with less than 1% among those without acute renal failure (p<0.01). Their risk factor analysis identified obesity with an odds ratio of 3.03, higher preoperative creatinine with an odds ratio of 4.21, and longer CPB time with an odds ratio of 1.06 per 10-minute increment as important predictors, showing that even incremental prolongation of bypass duration may carry renal consequences. A research from Pakistan also reported a significantly higher rate of postoperative AKI among patients with longer CPB time, although the absolute percentages were lower than those of the present study, with AKI occurring in 6.59% of patients with prolonged CPB compared with 1.1% in those with shorter CPB duration (p=0.050) [9]. This difference in magnitude may be explained by methodological and clinical heterogeneity between the studies.

## Conclusions

This study concluded that prolonged CPB time was significantly associated with a higher occurrence of early postoperative AKI among adult patients undergoing cardiac surgery. Although the two groups were largely comparable in baseline characteristics, including age, gender, comorbidities, BMI, and disease pattern, patients with longer CPB time showed greater postoperative renal stress than those with shorter CPB time, as reflected by higher serum creatinine, lower urine output, and a markedly increased frequency of AKI. This renal vulnerability was accompanied by a slower postoperative recovery, with longer mechanical ventilation, ICU stay, and postoperative hospital stay among patients exposed to prolonged CPB. The findings should be interpreted in light of some limitations, including the single-center design, convenience sampling, short follow-up limited to the early postoperative period and hospital discharge, and the possible influence of unadjusted perioperative factors such as diuretic use, surgical complexity, transfusion, aortic cross-clamp time, and other intraoperative variables. While this study does not establish a causal relationship, it suggests that prolonged CPB duration may serve as an important perioperative marker for identifying patients at higher risk of early postoperative renal dysfunction. Therefore, patients exposed to longer CPB time may benefit from closer renal monitoring, careful postoperative assessment, and appropriate perioperative renal-protective attention, while larger adjusted studies are needed to determine whether reducing avoidable CPB prolongation can improve renal outcomes.

## Figures and Tables

**Figure 1:**
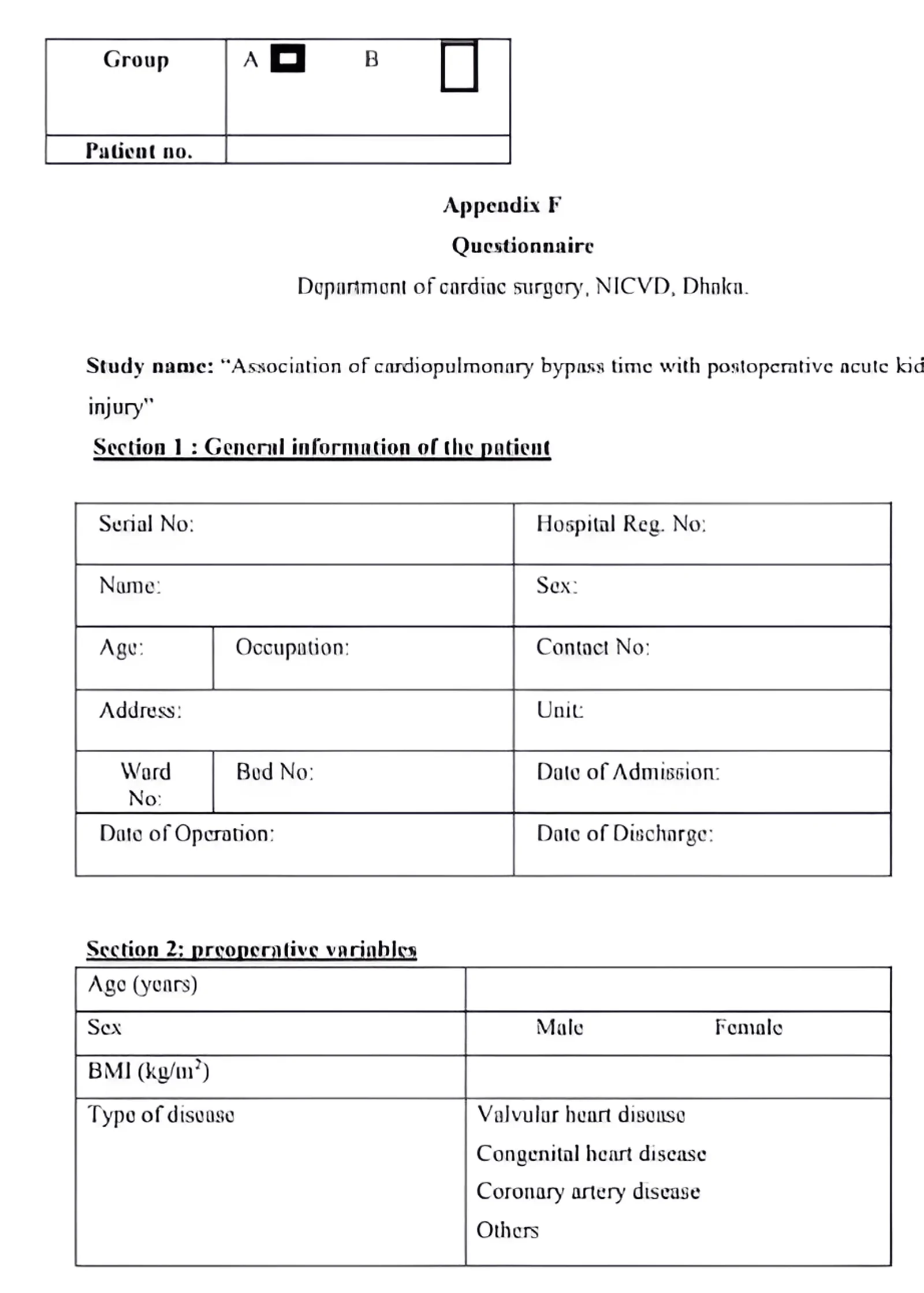
Questionnaire

**Figure 2:**
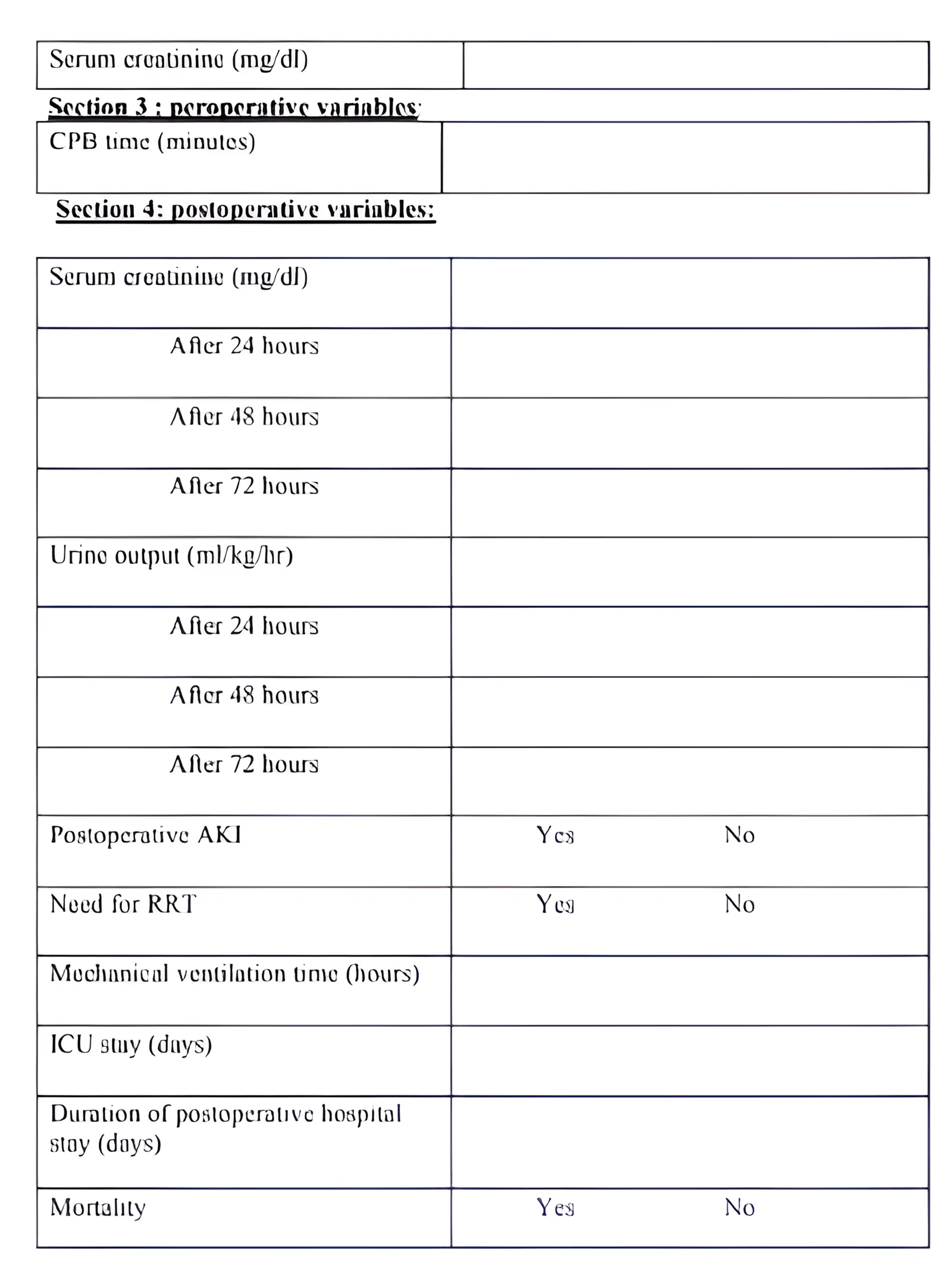
Questionnaire (continuation)

**Figure 3:**
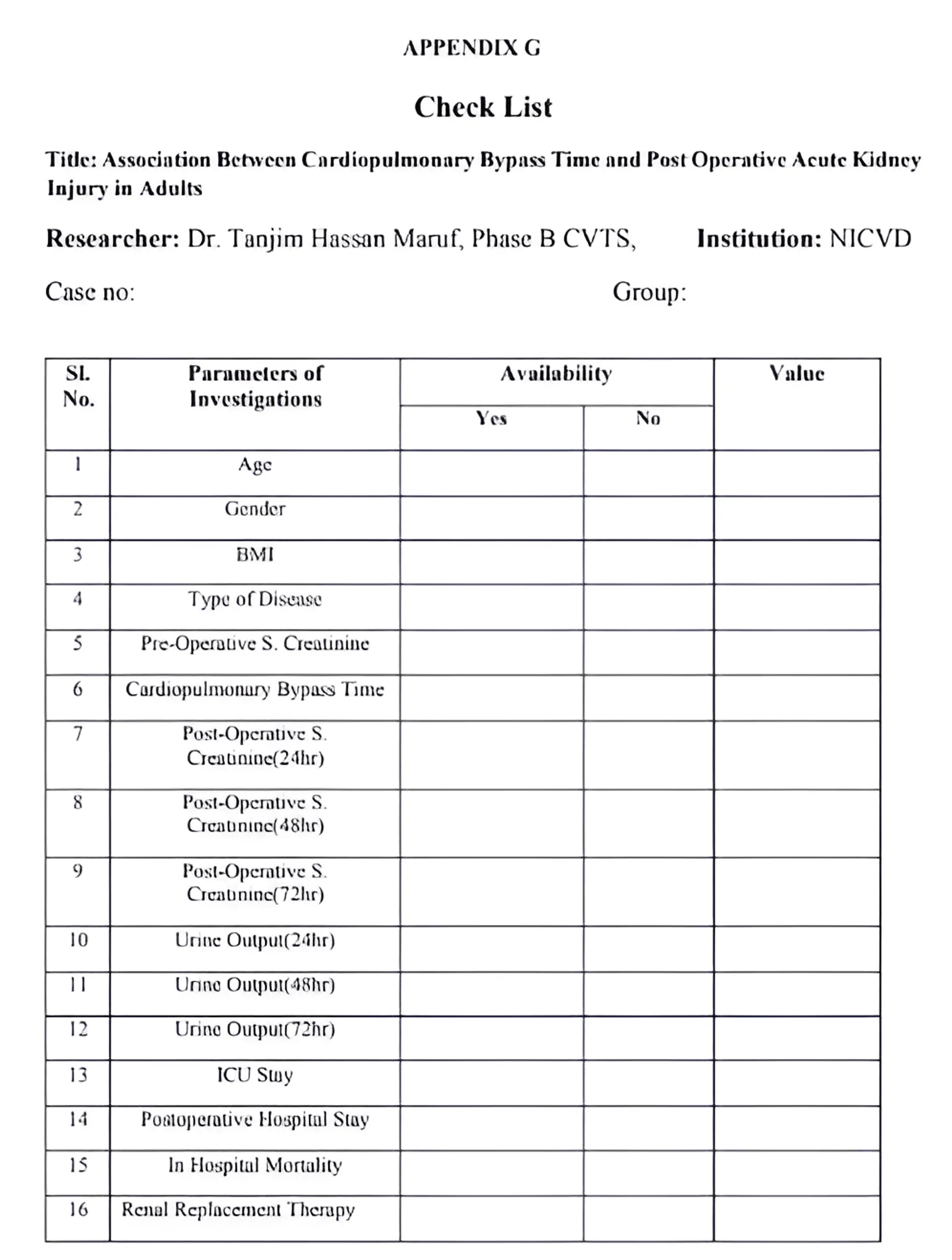
Checklist

## Appendices

Appendix A

Appendix B
